# The convergent roles of NF-κB and ER stress in sunitinib-mediated expression of pro-tumorigenic cytokines and refractory phenotype in renal cell carcinoma

**DOI:** 10.1038/s41419-018-0388-1

**Published:** 2018-03-07

**Authors:** Peter Makhov, Sei Naito, Miki Haifler, Alexander Kutikov, Yanis Boumber, Robert G. Uzzo, Vladimir M. Kolenko

**Affiliations:** 1 Cancer Biology Program, Fox Chase Cancer Center, Philadelphia, PA 19111 USA; 2 Division of Urologic Oncology, Department of Surgery, Fox Chase Cancer Center, Philadelphia, PA 19111 USA; 3 Department of Hematology/Oncology, Fox Chase Cancer Center, Philadelphia, PA 19111 USA

## Abstract

Renal cell carcinoma (RCC) is the most common form of kidney cancer. While cure remains exceptionally infrequent in RCC patients with systemic or recurrent disease, current targeted molecular strategies, including multi-targeted tyrosine kinase inhibitors (TKIs), notably changed the treatment paradigm of advanced renal cancer. Yet, complete and durable responses have been noted in only a few cases. Our studies reveal that sunitinib triggers two resistance-promoting signaling pathways in RCC cells, which emanate from the endoplasmic reticulum (ER) stress response: a PERK-driven ER stress response that induces expression of the pro-tumorigenic cytokines IL-6, IL-8, and TNF-α, and a TRAF2-mediated NF-κB survival program that protects tumor cells against cell death. PERK blockade completely prevents sunitinib-induced expression of IL-6, IL-8 and TNF-α, whereas NF-κB inhibition reinstates sensitivity of RCC cells to sunitinib both in vitro and in vivo. Taken together, our findings indicate that ER stress response may contribute to sunitinib resistance in RCC patients.

## Introduction

Renal cell carcinoma (RCC) is a lethal disease with rising incidence^1^. It is categorized into various subtypes, with clear cell RCC (ccRCC) representing about 75% of all RCC tumors^2^. Currently, there is no curative treatment for patients who present with metastatic disease or those who recur following definitive surgical therapy for localized ccRCC. Cure remains exceptionally rare in these patients. However, current targeted molecular strategies, including tyrosine kinase inhibitors (TKIs), have resulted in a doubling of progression-free survival and significant gains in overall survival (median 18–30 months), thereby fundamentally changing the treatment paradigm of advanced kidney cancer^3,4^. Unfortunately, about 21% of ccRCC patients are primarily refractory to the treatment with TKIs, showing neither disease stabilization nor clinical benefits^2^. Moreover, most patients that respond initially will typically progress within 12 months of starting therapy. Median overall survival in patients with metastatic ccRCC treated with TKIs remains in the region of 24 months^5,6^.

A family of NF-κB (nuclear factor kappa B) transcription factors functions as a key regulator of a variety of biological processes, including immunity, cell adaptation and survival, proliferation, and apoptosis^7^. Multiple studies have established the role of NF-κB regulated genes in malignant transformation, metastatic tumor progression and resistance to therapeutic regimens^8^. The aberrant activation of NF-κB results in upregulation of anti-apoptotic and pro-tumorigenic genes and promotes survival and migration of cancer cells^8,9^. A number of studies reported that constitutive NF-κB activity was observed in a variety of cancer types^10^. In addition, the activity of NF-κB may be induced by several stress factors including anticancer therapy^11^. A recent report by Tam et al. demonstrated a functional crosstalk between endoplasmic reticulum (ER) stress and activation of NF-κB^12^. ER functions include translation, modification and folding of secreted proteins. Misfolded proteins remain in the ER and are subjected to re-folding or degradation^13^. ER homeostasis may be disrupted by a variety of physiological and pathological stimuli resulting in accumulation of misfolded or unfolded proteins. Such accumulation, termed as ER stress, activates a cell signaling program, known as unfolded protein response (UPR), in order to restore ER homeostasis^14^. Activation of three types of ER stress sensors - protein kinase R (PKR)-like endoplasmic reticulum kinase (PERK), inositol-requiring enzyme 1 (IRE1α) and activating transcription factor 6 (ATF6)- by dissociation from ER chaperone, GRP78, induces the UPR^15^. Activated PERK phosphorylates translation initiation factor eIF2α, thus triggering suppression of protein translation. However, expression of ATF4 protein is increased upon activation of the PERK branch^16^. IRE1α represents the most evolutionary conservative branch of the UPR^17^. Activated IRE1α interacts with TRAF2, which results in downstream activation of c-Jun N-terminal kinase and NF-κB pathways^12^. In addition, an active RNAse domain of IRE1α exerts regulated IRE1α-dependent decay (RIDD of mRNA) activity. A transcription factor, X-box binding protein 1 (XBP1), which functions as for ER quality control genes, is generated by IRE1α-mediated processing of mRNA^18,19^. Activation of ATF6 depends on the dissociation from GRP78 and proteolytic cleavage. The cleaved ATF6 fragment translocates into the nucleus and upregulates transcription of target genes^20^. Recent studies demonstrate a link between ER stress and survival of cancer cells. The activation of pro-survival mechanisms by ER stress, such as an autophagy, may compromise the efficacy of anticancer therapy^21^. In contrast, persistent or severe ER stress results in apoptotic cell death^22^.

In the present study, we demonstrate, for the first time, that sunitinib triggers two resistance-promoting signaling pathways in ccRCC cells. These pathways emanate from the ER stress response: first, a PERK-driven ER stress response induces expression of the pro-tumorigenic cytokines interleukin-6 (IL-6), IL-8 and tumor necrosis factor-α (TNF-α). Second, a TRAF2-mediated NF-κB transcriptional survival program protects tumor cells against cell death. PERK blockade using pharmacological or genetic approaches completely prevents sunitinib-induced expression of IL-6, IL-8 and TNF-α, whereas NF-κB inhibition reinstates sensitivity of ccRCC cells to sunitinib both in vitro and in vivo. Our findings suggest that induction of ER stress may contribute to TKI resistance in RCC patients.

## Results

### Sunitinib induces NF-κB activation and augments expression of IL-6, IL-8 and TNF-α

**Fig. 1:**
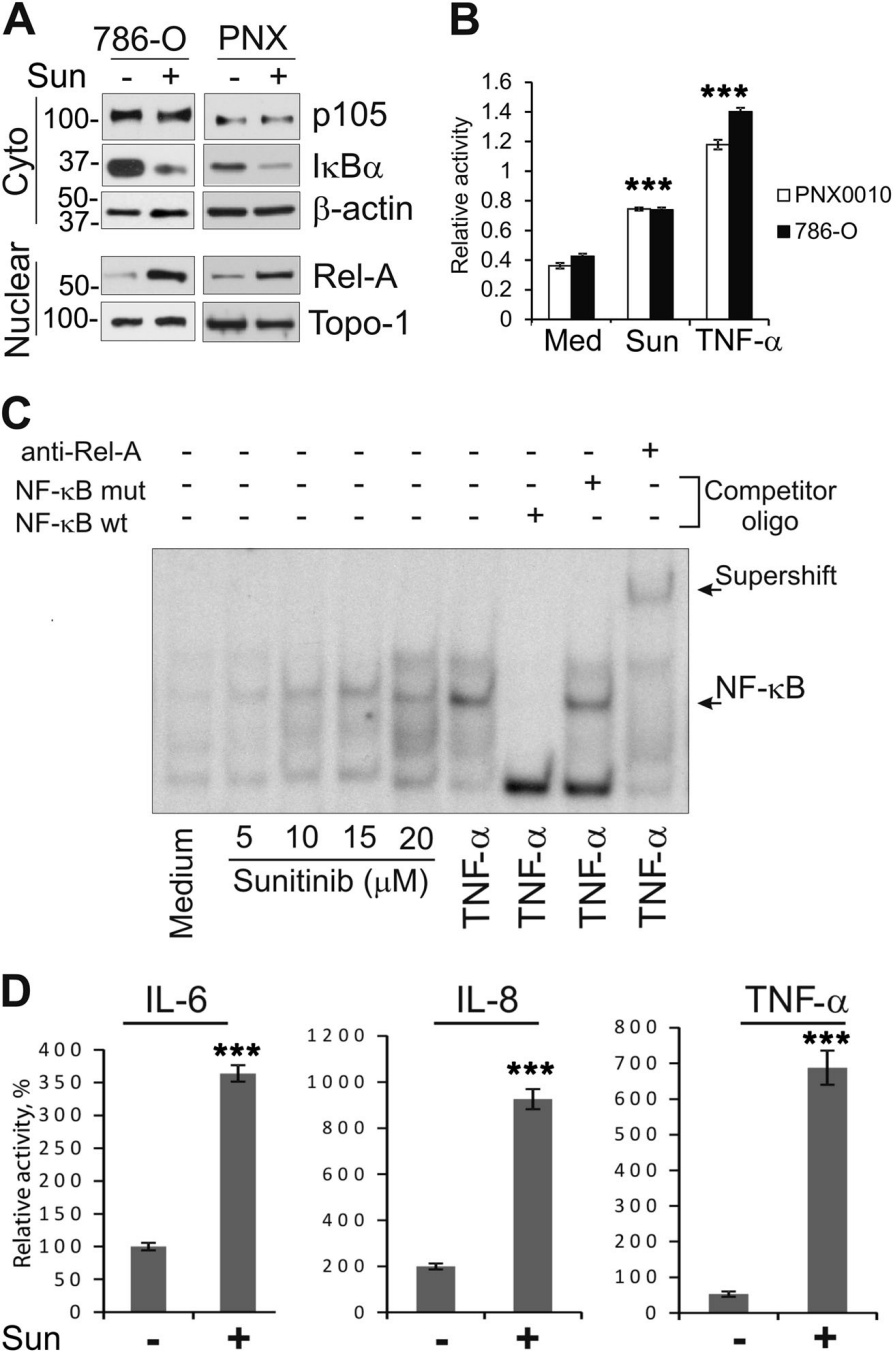
Treatment with sunitinib activates NF-κB and upregulates expression of IL-6, IL-8 and TNF-α in ccRCC cells. **a** Sunitinib (Sun) induces degradation of IκBα and promotes nuclear translocation of Rel-A in 786-O and PNX0010 ccRCC cells. Cells were treated with 10 μM of sunitinib for 3 h. Cytoplasmic and nuclear extracts were subjected to western blot analysis using specific antibodies. **b** 786-O and PNX0010 cells were treated with 10 μM of sunitinib for 4 h. NF-κB (p65) DNA binding activity was examined using TransAM assay. Data are presented as the mean ± S.D. ***P < 0.0001 when compared with cells cultured in medium only (Med). **c** 786-O cells were treated as described above. NF-κB activity was examined by EMSA in nuclear extracts prepared from 786-O cells. Supershift analysis using p50 antibody was performed to confirm that the observed bands are specific for NF-κB. **d** Sunitinib augments expression of IL-6, IL-8 and TNF-α in ccRCC cells. 786-O cells were treated with 10 μM of sunitinib for 10 h. The levels of IL-6, IL-8 and TNF-α were evaluated by ELISA (enzyme-linked immunosorbent assay). Data are presented as the mean ± S.D. ***P < 0.0005 when compared with cells cultured in medium only

In addition to inhibition of angiogenesis, TKIs also exert a direct cytotoxic effect on tumor cells^23–26^. Importantly, studies by Gotink et al. using human clinical specimens established that intratumor sunitinib levels are significantly higher than the corresponding plasma concentrations (9.5 ± 2.4 μmol/L vs. 0.3 ± 0.1 μmol/L, respectively)^23^. Recent studies have shown that resistance of tumor cells to various therapeutic agents could be driven, at least in part, by the augmented activation of NF-κB signaling in response to drug exposure^27^. We evaluated the level of NF-κB activity in established 786-O and patient-derived PNX0010 ccRCC cells treated with sunitinib. Treatment with sunitinib triggered the activation of NF-κB in both 786-O and PNX0010 RCC cells as assessed by western blot analysis of nuclear and cytoplasmic extracts. Sunitinib-induced degradation of the inhibitory subunit IκBα is accompanied by the nuclear translocation of Rel-A (Fig. 1a). Importantly, we did not observe any noticeable changes in NF-κB1 (p105) levels (Fig. 1a). These findings indicate that treatment with sunitinib promotes activation of canonical but not non-canonical NF-κB signaling. The results of western blot analysis were validated using TransAm assay (Fig. 1b) and EMSA (Fig. 1c). We carried out supershift assays to demonstrate that the observed bands are specific for NF-κB (Fig. 1c). Notably, sunitinib-mediated NF-κB activation coincided with the elevated expression of IL-6, IL-8 and TNF-α (Fig. 1d).

### Sunitinib-mediated activation of NF-κB depends on the IRE1α/TRAF2/IKKβ axis of the ER stress response

**Fig. 2:**
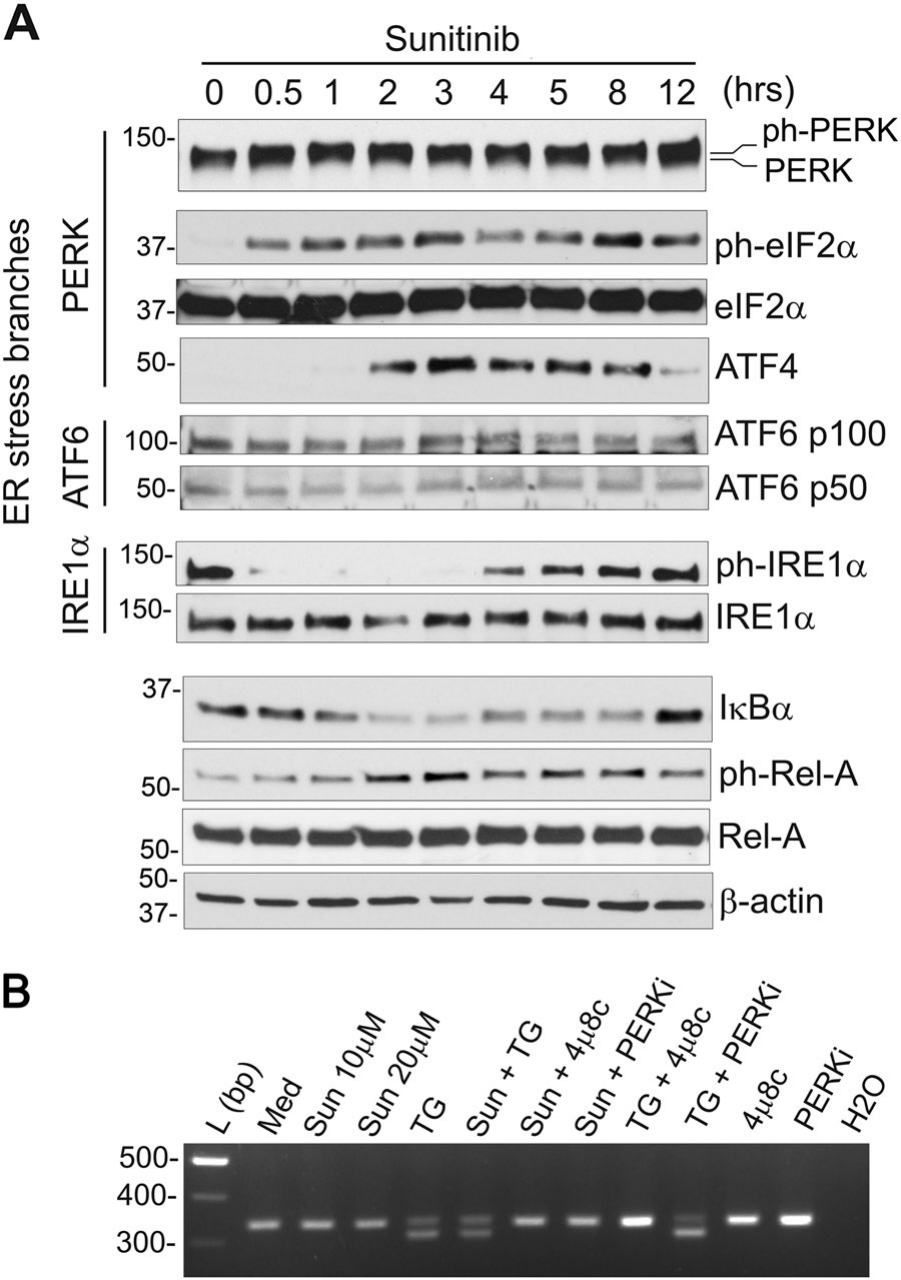
Sunitinib activates ER stress response in ccRCC cells. **a** Time-course of sunitinib-induced ER stress and NF-κB activation. 786-O ccRCC cells were treated with 10 μM of sunitinib for the indicated periods of time. Total cell lysates were subjected to western blot analysis using specific antibodies. **b** Treatment with sunitinib does not affect IRE1α-mediated Xbp1 splicing under ER stress conditions. 786-O cells were pre-treated with 5 μM of the PERK inhibitor GSK2656157 (PERKi) or 1 μM of the IRE1α inhibitor 4μ8c for 1 h followed by incubation in the presence or absence of sunitinib (10 μM) (Sun) or thapsigargin (1 μM) (TG) for 4 h. To explore whether treatment with sunitinib affects Xbp1 splicing under ER stress, 786-O cells were pre-incubated with sunitinib (10 μM) for 1 h followed by treatment with thapsigargin (1 μM) for 4 h. Total RNA was subjected to RT-PCR with specific primers spanning Xbp1 cleavage site. RT-PCR products were then separated in 2.5% agarose gel to detect spliced (lower band, 311 bp) and unspliced (upper band, 337 bp) fragments

Recent studies by Tam et al. indicate that activation of NF-κB may be triggered via ER stress response^12^. Therefore, we examined the potential contribution of the distinct branches of ER stress response in the regulation of sunitinib-mediated activation of NF-κB signaling. Sunitinib-induced NF-κB activation in 786-O RCC cells, as demonstrated by IκBα degradation and Rel-A phosphorylation, corresponds with the eIF2α phosphorylation, upregulation of ATF4 protein expression and dephosphorylation of IRE1α (Fig. 2a). Treatment with sunitinib and thapsigargin, a well-established ER stress inducer, in the presence or absence of either PERK inhibitor GSK2656157 or IRE1α inhibitor 4μ8C did not affect the expression level of Rel-A protein in 786-O cells (Supplementary Fig. 1). We also observed no changes in full-length and cleaved ATF6 protein levels (Fig. 2a), suggesting that the effect of sunitinib may be restricted to the IRE1α and PERK arms.

Recent studies demonstrate that sunitinib inhibits autophosphorylation of IRE1α, but may stabilize the active conformation of IRE1α ATP-binding site^28,29^. Our studies support these findings demonstrating that sunitinib does not inhibit RNAse activity of IRE1α under ER stress conditions (Fig. 2b).

**Fig. 3:**
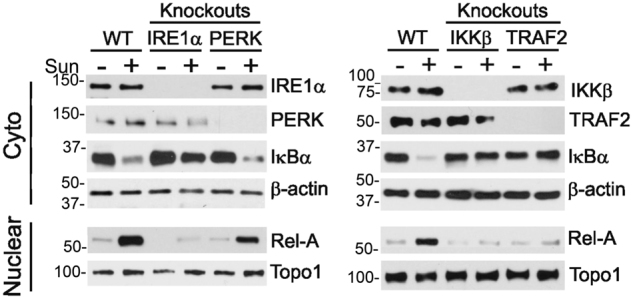
Sunitinib-mediated NF-κB activation depends on IRE1α/TRAF2/IKKβ branch of ER stress. Wild type (WT) 786-O cells and their knockout counterparts were treated with 10 μM of sunitinib (Sun) for 3 h. Cytoplasmic and nuclear extracts were subjected to western blot analysis using specific antibodies

In order to further substantiate our findings, we generated IRE1α, PERK and TRAF2 knockout sub-lines of 786-O cells using CRISPR/Cas9 technology. 786-O cells with IKKβ knockout were used as a control. As expected, knockout of IKKβ completely abolished sunitinib-induced NF-κB activation (Fig. 3). Also, no activation of NF-κB was detected in 786-O-IRE1α and -TRAF2 knockout cell lines treated with sunitinib. In contrast, knockout of PERK had no effect on sunitinib-induced NF-κB activation (Fig. 3), indicating that PERK arm of the ER stress response is not involved in the regulation of the NF-κB activation under these conditions.

### Critical roles of NF-κB and PERK branch of ER stress in sunitinib-mediated expression of pro-tumorigenic IL-6, IL-8 and TNF-α cytokines

**Fig. 4:**
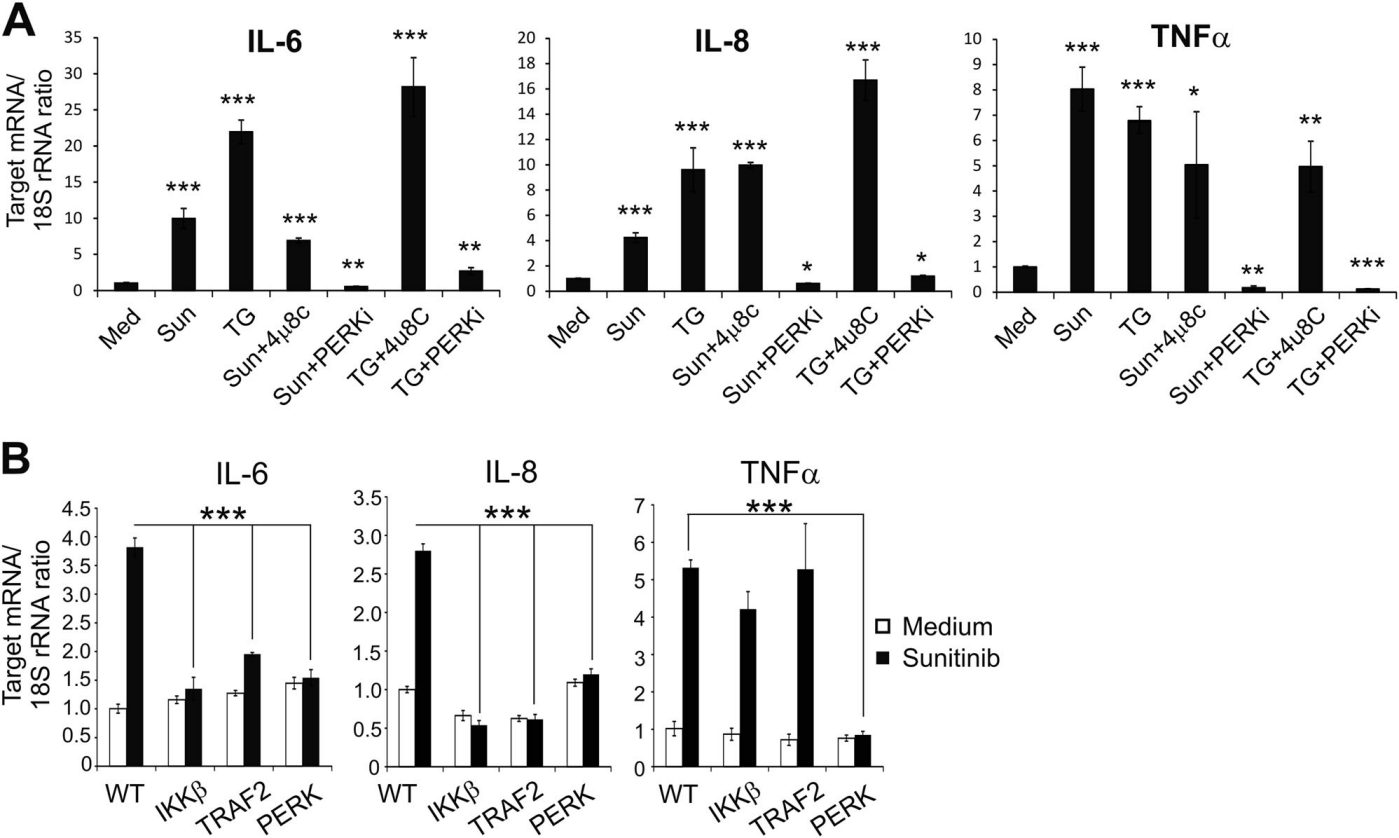
Sunitinib-induced expression of IL-6, IL-8 and TNFα depends on activation of PERK branch of ER stress. **a** Induction of ER stress in ccRCC cells results in upregulation of IL-6, IL-8 and TNFα expression. 786-O cells were pre-treated with 5 μM of the PERK inhibitor, GSK2656157 (PERKi) or 1 μM of the IRE1α inhibitor 4μ8 C for 1 h followed by incubation in the presence or absence of sunitinib (10 μM) (Sun) or thapsigargin (1 μM) (TG) for 4 h. Cytokines expression levels were analyzed by qRT-PCR. Data are presented as the mean ± S.D. *P < 0.05; **P < 0,001; ***P < 0.0005 when compared with cells cultured in medium only (Med). **b** The effects of IKKβ, TRAF2 and PERK knockouts on sunitinib-induced cytokine production in 786-O cells. Wild-type (WT) 786-O cells and their knockout counterparts were treated with 10 μM of sunitinib for 3 h. Cytokines expression levels were analyzed by qRT-PCR. Data are presented as the mean ± S.D. ***P < 0.0005

Both sunitinib and thapsigargin notably upregulated the expression of IL-6, IL-8 and TNF-α in 786-O cells (Fig. 4a). Pretreatment with a selective PERK inhibitor, GSK2656157, completely suppressed both thapsigargin and sunitinib-induced expression of all tested cytokines. In contrast, the addition of IRE1α inhibitor 4μ8C did not affect the expression levels of IL-6, IL-8 and TNF-α (Fig. 4a). These findings indicate that augmented expression of these molecules is mediated through the PERK branch of the ER stress.

Studies with IKKβ, TRAF2 and PERK knockout sub-lines of 786-O cells demonstrated that activation of both NF-κB and PERK was essential for sunitinib-induced production of IL-6 and IL-8 in tumor cells (Fig. 4b). In contrast, the expression of TNF-α was dependent on PERK activity only (Fig. 4b). These findings suggest that both NF-κB and PERK may serve as attractive therapeutic targets to modulate TKI-induced cytokine response in RCC tumors.

### Activation of NF-κB promotes survival of RCC cells during sunitinib treatment

**Fig. 5:**
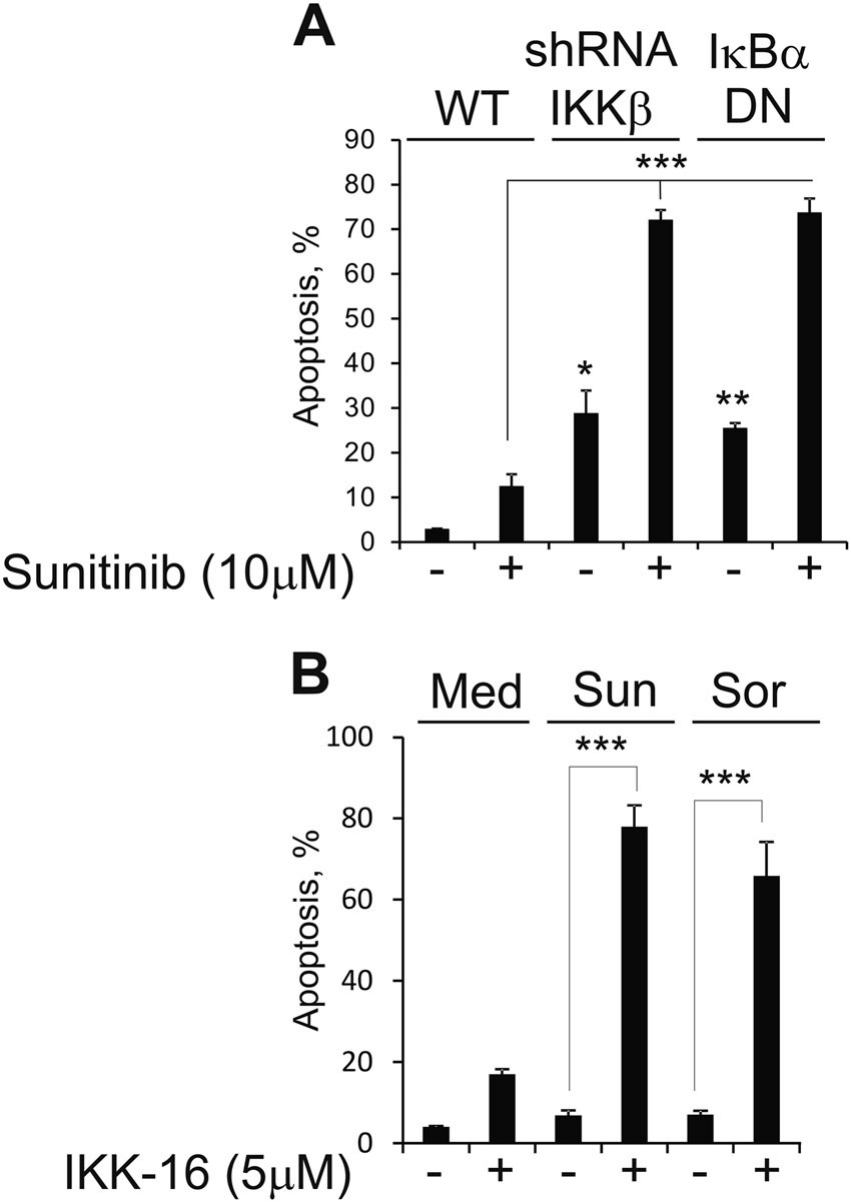
NF-κB activation promotes survival of ccRCC cells treated with sunitinib. **a** Wild type (WT) 786-O cells, IKKβ knockout subline and cells expressing HA-tagged dominant-negative mutant IκBα (S32A/S36A) were treated with 10 μM of sunitinib for 24 h. Apoptosis was examined using APO-BRDU kit followed by flow cytometry analysis. Data are presented as the mean ± S.D. *P = 0.0002; **P < 0.0001; ***P < 0.0001 when compared with WT 786-O cells treated with sunitinib. **b** 786-O cells were treated with either 10 μM of sunitinib (Sun) or 10 μM of sorafenib (Sor) for 24 h with or without IKKβ inhibitor, IKK-16 (5 μM). Apoptosis was examined using APO-BRDU kit followed by flow cytometry analysis. Data are presented as the mean ± S.D. ***P < 0.0001.

**Fig. 6:**
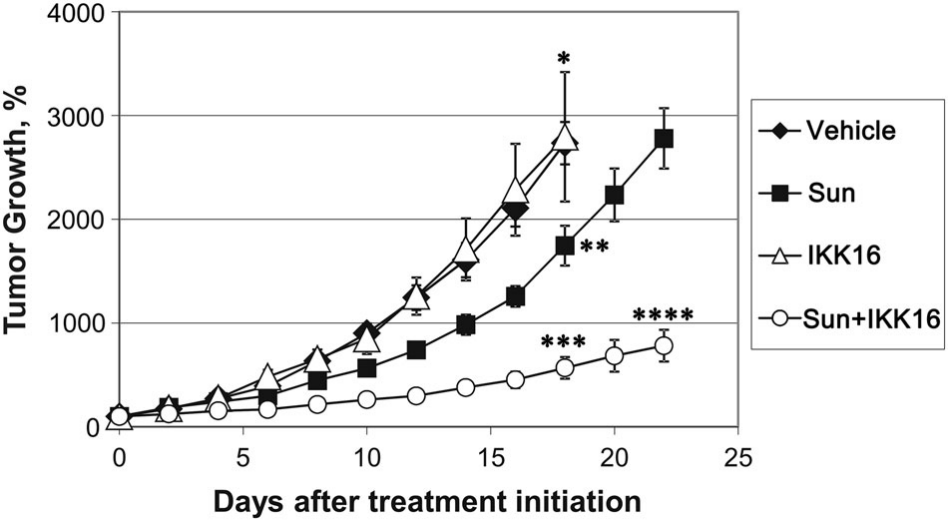
Treatment with IKK-16 overcomes resistance to sunitinib in a xenograft animal model of human ccRCC. Subcutaneous PNX0010 tumors were established in 6-week-old male C.B17icr-scid mice. Treatment with sunitinib (Sun), IKK-16 or combination was carried out as described in Materials and methods section. Values are means (*n* = 5) ±SEM. *P = 0.0069 when compared vehicle with sunitinib treatment; **P < 0.0001 when compared sunitinib with sunitinib + IKK-16 treatment; ***P < 0.0001 when compared vehicle with sunitinib + IKK-16 treatment; ****P < 0.0001 when compared sunitinib with sunitinib + IKK-16 treatment at the end of the experiment.

Short hairpin RNA-mediated knockdown of IKKβ or expression of dominant-negative (DN) IκBα reinstated sensitivity of 786-O cells to sunitinib-induced apoptosis (Fig. 5a). Furthermore, treatment with IKKβ inhibitor, IKK-16, also overcame resistance to sunitinib and sorafenib in 786-O cells (Fig. 5b). Interestingly, the results of quantitative reverse transcriptase-PCR (qRT-PCR) analysis did not reveal substantial changes in the expression levels of pro- and anti-apoptotic Bcl-2 family members in sunitinib-treated 786-O cells (Supplementary Fig. 2). Taken together, our results suggest that NF-κB activation is essential for survival of RCC cells upon TKIs treatment, whereas PERK activity is critical for the production of pro-tumorigenic cytokines by tumor cells. To corroborate our in vitro finding, we examined the effect of concomitant treatment with IKK-16 and sunitinib on tumor growth using PNX0010 RCC xenograft model. The combined treatment with IKK-16 and sunitinib notably inhibited growth of PNX0010 tumors (Fig. 6). Importantly, this treatment was well tolerated by all experimental animals. These data support our hypothesis that NF-κB contributes to the activation of intrinsic resistance mechanisms promoting RCC cell survival during TKI treatment.

## Discussion

There are two fundamental mechanisms underlining resistance of RCC to TKIs. First, extrinsic mechanisms bypass TKI-mediated antiangiogenic activity by providing alternative means for TKI-resistant intra-tumoral blood vessel growth. For instance, production of alternative pro-angiogenic factors, such as fibroblast growth factors (FGFs) and placenta growth factor (PlGF), or downregulation of angiostatic chemokines, such as Cxcl9, or induction of pro-inflammatory cytokines, that is, IL-1, IL-6, TNF-α, by tumor cells have been implicated in the mechanisms of TKI resistance^30–32^. Second, intrinsic mechanisms of TKI resistance include activation of alternative pro-survival signaling pathways within the tumor cells that make these cells refractory to TKI-mediated death. NF-κB is one of the most important cell survival pathways known to play a critical role in resistance to chemo- and radiotherapy in various solid and hematologic malignancies^33–35^.

**Fig. 7:**
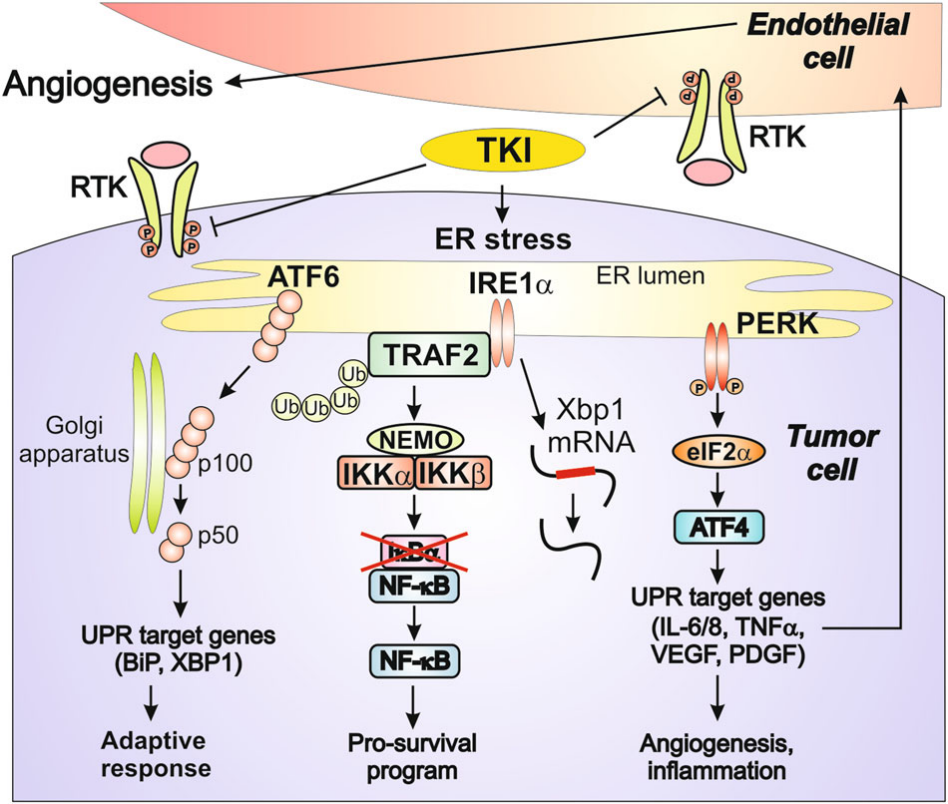
Potential mechanisms of TKI-mediated activation of ER stress and NF-κB signaling

Along with others, we have demonstrated that, in addition to the inhibition of angiogenesis, sunitinib can exert a direct cytotoxic effect on tumor cells^23–26^. Our current findings indicate that sunitinib triggers two resistance-promoting signaling pathways in RCC cells, both of which emanate from the ER stress response: a PERK-driven ER stress response that induces expression of the pro-tumorigenic cytokines IL-6, IL-8 and TNF-α, and a TRAF2-mediated NF-κB transcriptional survival program that protects tumor cells against cell death (Fig. 7). Potentially, ER stress may activate NF-κB, at least in part, indirectly via autocrine TNF-α production. However, this possibility can be excluded based on our findings showing that knockout of PERK does not affect sunitinib-mediated NF-κB activation in 786-O cells (Fig. 3), despite the fact that PERK knockout or pretreatment of cells with PERK inhibitor, GSK2656157 (Figs. 4a, b) results in complete inhibition of TNF-α production.

Similar to data obtained by Tam et al., our experiments demonstrate that sunitinib-mediated activation of NF-κB depends on IRE1α branch of ER stress. Indeed, knockout of IRE1α dramatically affects NF-κB activity induced by sunitinib^12^. Our studies indicate that IRE1α-dependent activation of NF-κB in sunitinib-treated cells occurs concomitantly with reduction of IRE1α phosphorylation. Normally, IRE1α’s RNAse activity depends on its kinase trans-autophosphorylation^36^. However, sunitinib may stimulate IRE1α RNAse activity independently of its trans-autophosphorylation by stabilizing the active conformation of its ATP-binding site^28,29^. Our data indicate that sunitinib suppressed IRE1α kinase activity, but did not affect its RNAse activity. We therefore hypothesize that, in spite of the negative effect of sunitinib on IRE1α phosphorylation, sunitinib stabilizes its active conformation, thereby facilitating interaction with TRAF2 resulting in IKKβ-dependent activation of NF-κB.

It was reported previously that treatment with TKIs results in upregulation of pro-angiogenic and pro-inflammatory cytokines, IL-6, IL-8 and TNF-α^24,32,37^. Our current study demonstrates that, in addition to the well-established NF-κB-dependent regulation of IL-6 and IL-8 expression, PERK activity is also critical for the augmented expression of these cytokines upon treatment with sunitinib. Our data indicate that the sunitinib-induced expression of TNF-α depends on PERK activity only. Indeed, downregulation of NF-κB activation via TRAF2 or IKKβ knockout failed to prevent sunitinib-mediated upregulation of TNF-α expression. However, treatment with a selective PERK inhibitor, GSK2656157, completely suppressed both thapsigargin- and sunitinib-mediated expression of IL-6, IL-8 and TNF-α. Our data are in strong agreement with the recent study by Wang et al. demonstrating that PERK/ATF4 branch of ER stress controls angiogenic switch in human cancer cells in response to glucose deprivation^38^. Therefore, PERK could serve as an attractive therapeutic target to modulate TKI-induced cytokine response.

Our study demonstrates for the first time that sunitinib activates NF-κB signaling via IRE1α-mediated mechanism. Knockout of IRE1α completely blocked sunitinib-induced NF-κB activation in 786-O cells (Fig. 3). In addition, our findings indicate that, although PERK activity is essential for sunitinib-mediated expression of IL-6, IL-8 and TNF-α, such activity is dispensable for cell survival. Indeed, pretreatment of 786-O cells with PERK inhibitor GSK2656157 failed to sensitize cells to sunitinib (data not shown). A recent study by Han et al. suggested pro-survival effect of PERK/eIF2α branch of ER stress pathway^39^. The authors reported that BiP/GRP78 depletion results in decreased PERK phosphorylation and suppression of activity of its downstream target, eIF2α. However, these findings are in strong controversy with general biological functions of BiP/GRP78. This chaperone acts as the main binding partner of three ER sensors, specifically ATF6, IRE1α and PERK, keeping them inactive in sequestration^40,41^. Moreover, a number of publications documented that depletion of BiP/GRP78 results in activation of ER stress pathways, including PERK branch^42,43^.

The current study demonstrates for the first time that sunitinib induces two resistance-promoting signaling pathways, both of which originate from the ER stress response: a PERK-driven pathway that regulates the expression of IL-6, IL-8 and TNF-α cytokines, and a TRAF2-mediated NF-κB pro-survival pathway. Taken together, our findings indicate that ER stress response may contribute to sunitinib resistance in RCC patients.

## Materials and methods

### Cells and culture conditions

The 786-O human ccRCC cells (CRL-1932) and the 293T human embryonic kidney cells (CRL-3216) were obtained from ATCC (Rockville, MD). Authentication of 786-O and 293T cells were performed by IDEXX BioResearch (West Sacramento, CA). The PNX0010 patient-derived ccRCC cell line^44^ was a kind gift of Vladimir Khazak, Ph.D. (Priaxon Inc., Philadelphia, PA). Initial stocks were cryopreserved, and at every 6-month interval a fresh aliquot of frozen cells was used for the experiments. Cells were cultured in RPMI-1640 (Bio-Whittaker, Walkersville, MD) supplemented with 10% FBS (Hyclone, Logan, UT), penicillin (100 U/ml), streptomycin (100 μg/ml), sodium pyruvate (1 mM) and non-essential amino acids (0.1 mM) under conditions indicated in the figure legends.

### Antibodies and reagents

Antibodies to Rel-A (#8242), phospho-Rel-A (S536) (#3033), NF-κB1 (p105/p50) (#13586), TRAF2 (#4712), IKKβ (#8943), IRE1α (#3294), PERK (#5683), eIF2α (#5234), phospho-eIF2α (S51) (#9721) and ATF4 (#11815) were obtained from Cell Signaling Technology (Beverly, MA). ATF6 (sc-22799), Topo-1 (sc-32736), IκBα (sc-203), β-actin (sc-130300) and HA-probe (sc-7392) antibodies were obtained from Santa Cruz Biotechnology Inc. (Santa Cruz, CA). Phospho-IRE1α (S724) (#GTX63722) antibody was obtained from GeneTex (Irvine, CA). IKK-16 (#S2882) and GSK2656157 (#S7033) were obtained from Selleckchem (Radnor, PA). Sunitinib (#S-8803) was obtained from LC Laboratories (Woburn, MA). TNF-α (#CYT-223) was obtained from ProSpec (East Brunswick, NJ). 4μ8C (#412512) was obtained from Calbiochem (Burlington, MA).

### Western blot analysis

Cell lysates preparation and western blot analysis were performed as described previously^45^.

### ESMA and Gel-Supershift assays

ESMA and Gel-Supershift assays were performed as described previously^46^.

### qRT-PCR and Xbp1 splicing analysis

Total RNA was isolated using Quick-RNA™ MiniPrep (#R1054) (Zymo Research, Orange, CA) following DNase I (#M0303S) (New England Biolabs, Ipswich, MA) treatment. Then, RNA was purified using RNA Clean and Concentrator Kit (#R1015) (Zymo Research, Orange, CA). Total RNA (1 μg) was reverse transcribed in final volume of 20 μl with 100 U of Superscript III Reverse Transcriptase (#18080-051) (Life Science, Gaithersburg, MD) and 75 ng of random hexamer primers according to the manufacturer’s instructions. After reverse transcription, complementary DNA (cDNA) samples were diluted 40 times and 5 μl of diluted cDNA were amplified by real-time PCR using TNF-α TaqMan Gene Expression Assay (ID# Hs.PT.58.45380900), IL-6 TaqMan Gene Expression Assay (ID# Hs.PT.53a.24948568), IL-8 TaqMan Gene Expression Assay (ID# Hs.PT.56a.39926886.g), Bcl-2 TaqMan Gene Expression Assay (ID# Hs.PT.56a.654557.g), Bcl-2A1 TaqMan Gene Expression Assay (ID# Hs.PT.56a.1995943), BIM TaqMan Gene Expression Assay (ID# Hs.PT.58.27975199) and Mcl1 TaqMan Gene Expression Assay (ID# Hs.PT.58.1431437). Custom 18S rRNA Mini qPCR assay was used as internal amplification control. The amplicon was detected with a forward primer 5′-GCTCTTTCTCGATTCCGT-3′, reverse primer 5′-CCAGAGTCTCGTTCGTTATC-3′ and probe 5′-TTCTTAGTTGGTGGAGCGATTTGT-3′ labeled with 6-FAM and quenched with Jowa-Black FQ. All assays and primers were obtained from IDT DNA Technologies (Coralville, IA). Each sample was run in triplicate for each gene in a 20 μl reaction using TaqMan Gene Expression Master Mix according to the manufacturer’s instructions (#4369016) (Applied Biosystems, Foster City, CA). Reactions were carried out in an Applied Biosystems 7500 Real-Time PCR System. Relative expression was analyzed using the 2^−ΔΔCt^ method^47^.

For Xbp1 splicing analysis, 1 μl of reverse transcription reaction mixture was amplified with specific primers spanning Xbp1 cleavage site (forward 5′-AGCAAGTGGTAGATTTAGAAGA-3′ and reverse 5′- GTCCAAGTTGTCCAGAATG-3′). RT-PCR products were then separated in 2.5% agarose gel to detect spliced (311 bp) and unspliced (337 bp) fragments.

### Generation of CRISPR/Cas9-mediated knockout cell lines

CRISPR/Cas9-mediated knockout was performed using pLentiCRISPRv2 lentiviral vector^48^ (Addgene plasmid # 52961, a gift from Feng Zhang). Double-stranded DNA oligos (Supplementary materials, Table 1) targeting the desired genomic region were cloned into pLentiCRISPRv2 following the protocol provided by F. Zhang laboratory (available online at www.addgene.org). 293T cells were concomitantly transfected with following plasmids, pLentiCRISPRv2, psPAX2 (Addgene plasmid # 12260) and pVSV-G (Clontech, Mountain View, CA) using TransIT-2020 Transfection reagent (MIR 5400) (Mirus, Madison, WI). Supernatant of 293T cells containing lentiviral particles was collected 48 h after transfection and filtered through 0.22 μm PVDF syringe filters. The infection of 786-O cells was performed in 10 cm dishes in presence of 4 μg/ml Polybrene (Hexadimethrine bromide) in 9 ml of cellular media by addition of 1 ml of 293T filtered supernatant. Forty-eight hours after infection, puromycin (1 μg/ml) was added to the cellular media for further selection of infected cells. After 10–14 days, cells were analyzed by western blotting for the knockouts efficacy.

### Generation of cells expressing DN IκBα

 Open Reading Frame of DN IκBα (S32A/S36A) was amplified using pCMV-IκBα(S32A/S36A)-HA vector (a kind gift from N. Dulin, Ph.D., University of Chicago, IL) as a template and re-cloned into pLV-CMV-H4-puro lentiviral construct. Generation of lentiviral particles and infection of 786-O cells was performed as described previously^49^.

### Analysis of apoptosis

DNA fragmentation was detected using APO-BRDU kit (#AU1001) (The Phoenix Flow Systems, Inc., San Diego, CA) as described previously^50^.

### Measurement of IL-6, IL-8 and TNF-α proteins

IL-6, IL-8 and TNF-α protein levels in cell culture supernatants were determined by ELISA kits (#D6050, #D8000C and #DTA00C, respectively) (R&D Systems, Minneapolis, MN).

### Assessment of in vivo tumor growth

For in vivo studies, 2 × 10^6^ of PNX0010 cells were injected subcutaneously in the flank region of 6-week-old male C.B17/icr-scid mice using a 27-gauge needle. All animal procedures were done in accordance with institutional guidelines on animal care and with appropriate institutional certification. Animals were fed sterile AIN-93M diet (Harlan Teklad, Madison, WI) and water ad libitum. Ten days after the injection of tumor cells, animals were randomly assigned to the control or experimental groups (*n* = 5 mice/group). The mice were treated daily with (i) 0.15 M NaCl with 2% dimethylsulfoxide (DMSO) solution (vehicle); (ii) sunitinib malate (20 mg/kg in 0.15 M NaCl with 2% DMSO solution, P.O.); (iii) IKK-16 (10 mg/kg, in 0.15 M NaCl with 2% DMSO, I.P.); or (iv) sunitinib and IKK-16 combined. Tumors were measured twice weekly and their volumes were calculated by the formula: [volume = 0.52 × (width)^2^ × length].

### Statistical analysis

Continuous variables are presented as mean (±SD). Comparison of continuous variables was performed using a two-sided Student’s* t*-test. A *P*-value of <0.05 was considered statistically significant.

## Electronic supplementary material

Supplementary Table 1

Supplementary Figure 1

Supplementary Figure 2
