# Supplementary material for: The convergent roles of NF-κB and ER stress in sunitinib-mediated expression of pro-tumorigenic cytokines and refractory phenotype in renal cell carcinoma
Source: Cell Death Dis. 2018 Mar 7;9(3):374. doi: 10.1038/s41419-018-0388-1 (PMC5841329; doi:10.1038/s41419-018-0388-1)
Supplement: Supplementary file 1 — Supplementary Table 1 [file 41419_2018_388_MOESM1_ESM.docx]

| **Gene name** | **Targeting gRNA oligo** |
| --- | --- |
| IRE1α | 5`- cgctgctgctgcccggcctc |
| PERK | 5`- cgcggcaaggacggtggccg |
| TRAF2 | 5`- cggcttctccaagaccctcc |
| IKKβ | 5`- cagtctttgcacatcattcg |

Supplementary Table 1. sgRNA targeting oligo sequences used to generate CRISPR/Cas9 mediated knockouts of indicated genes.
