# Supplementary figures and images for: The convergent roles of NF-κB and ER stress in sunitinib-mediated expression of pro-tumorigenic cytokines and refractory phenotype in renal cell carcinoma
Source: Cell Death Dis. 2018 Mar 7;9(3):374. doi: 10.1038/s41419-018-0388-1 (PMC5841329; doi:10.1038/s41419-018-0388-1)

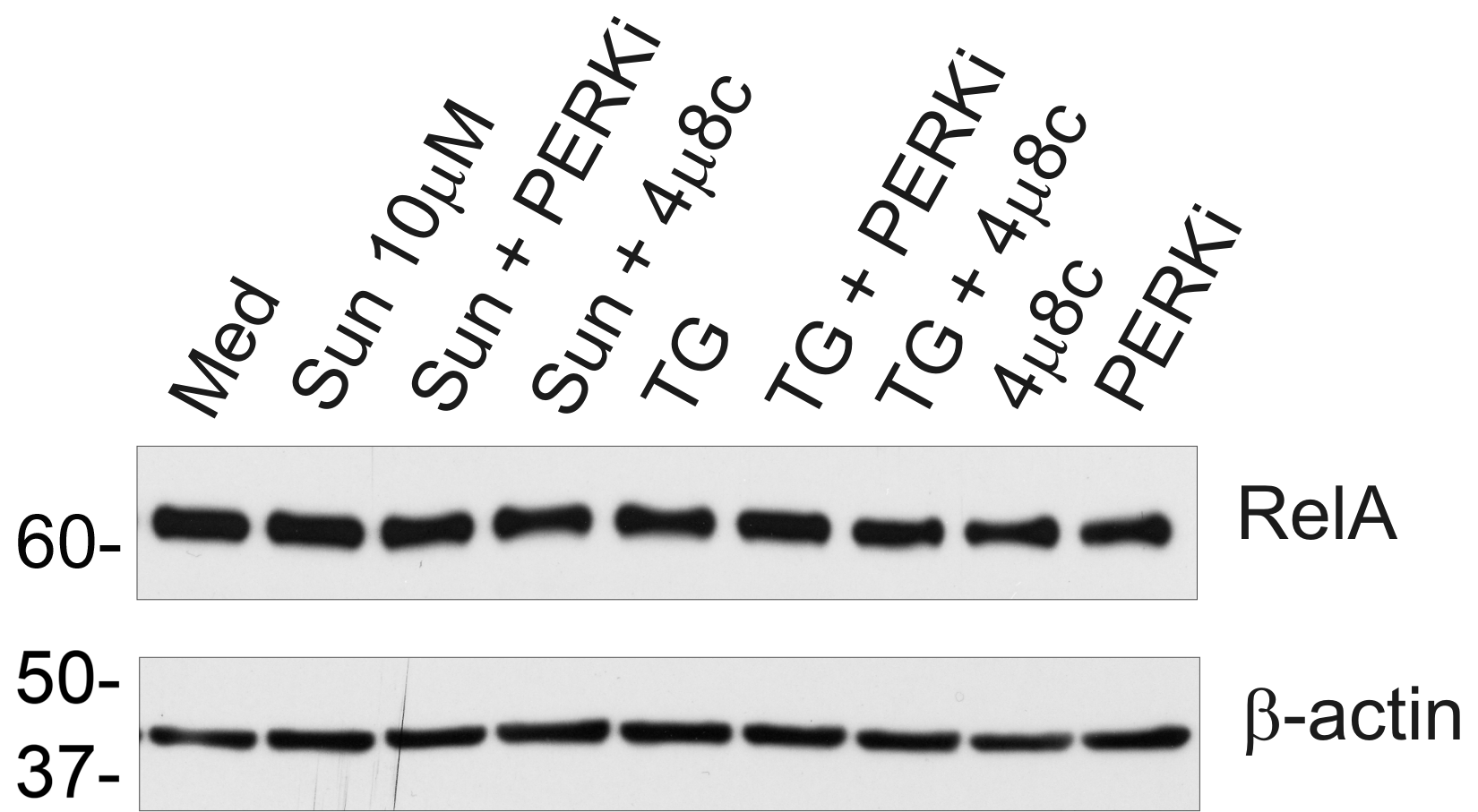

Supplement: Supplementary file 2 — Supplementary Figure 1 [file 41419_2018_388_MOESM2_ESM.tif]

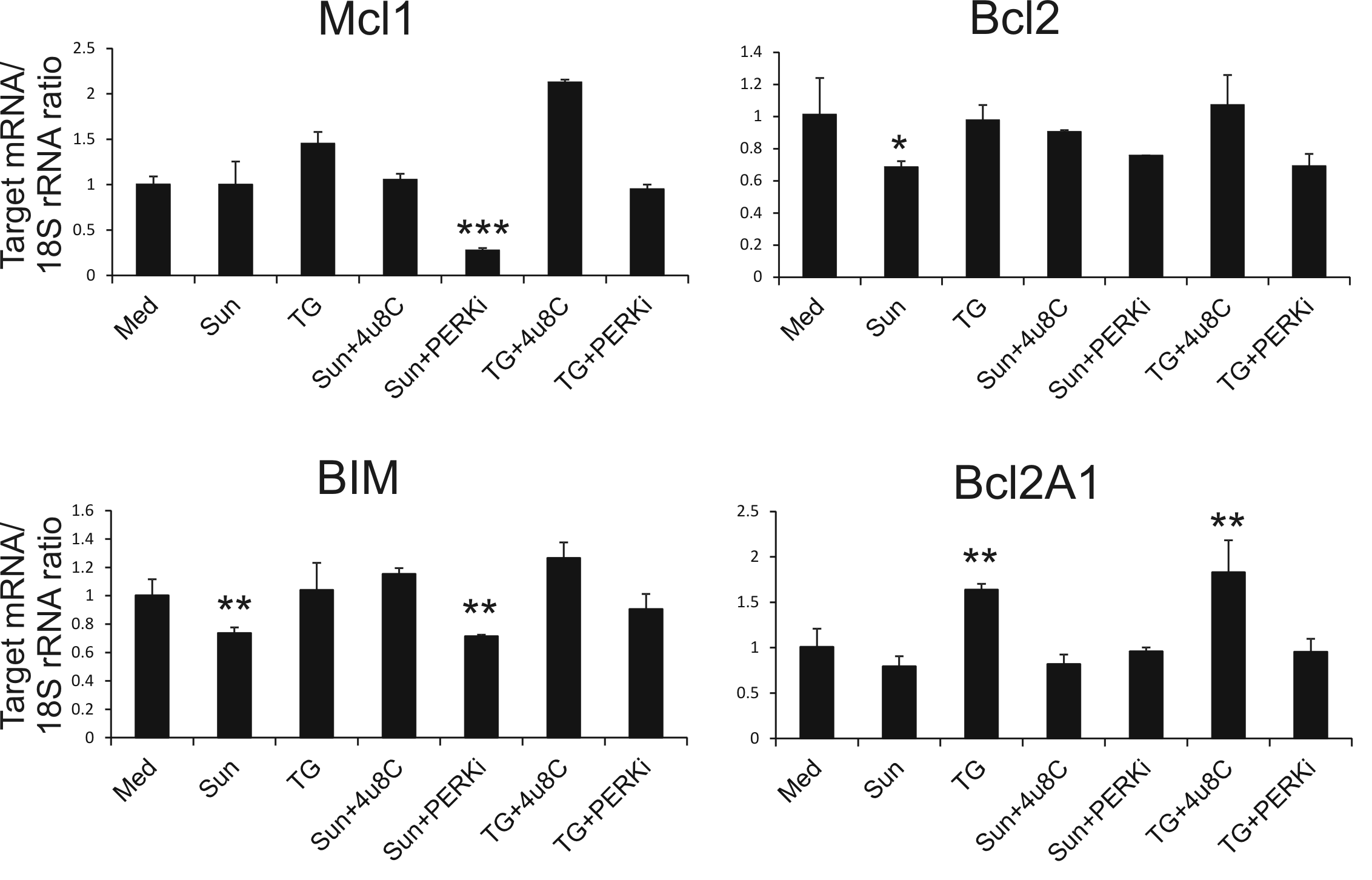

Supplement: Supplementary file 3 — Supplementary Figure 2 [file 41419_2018_388_MOESM3_ESM.tif]
